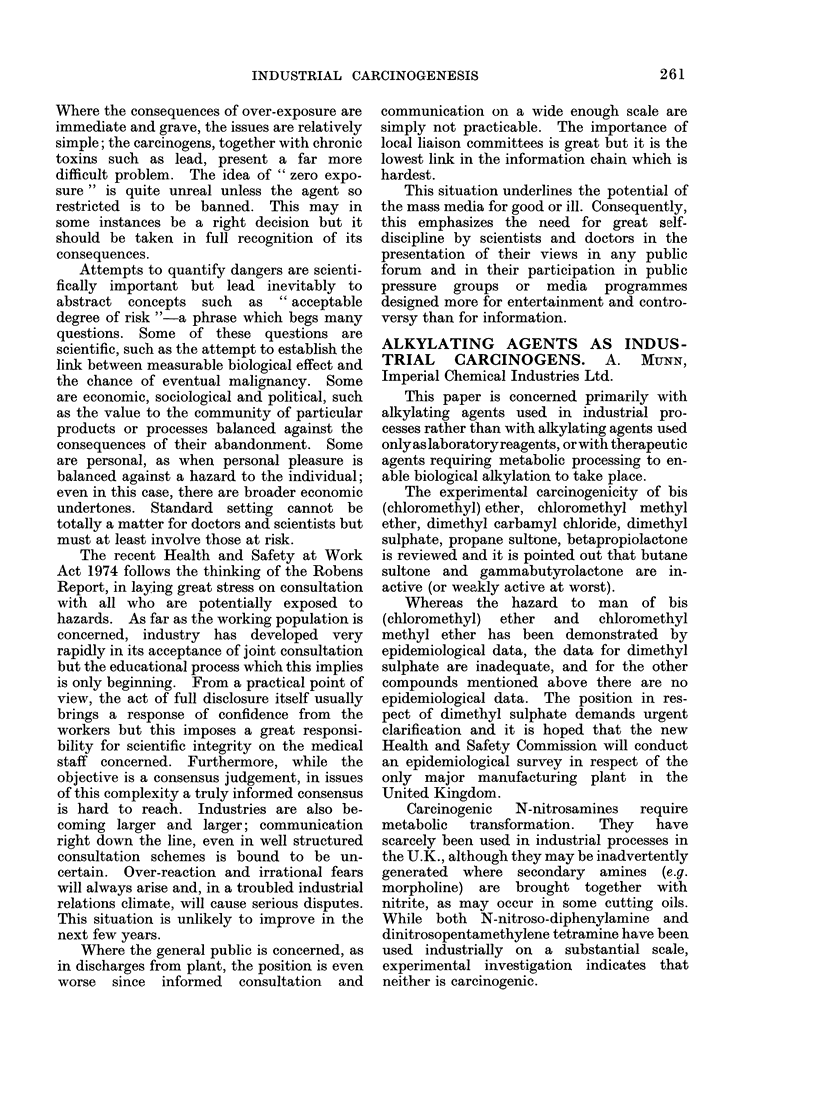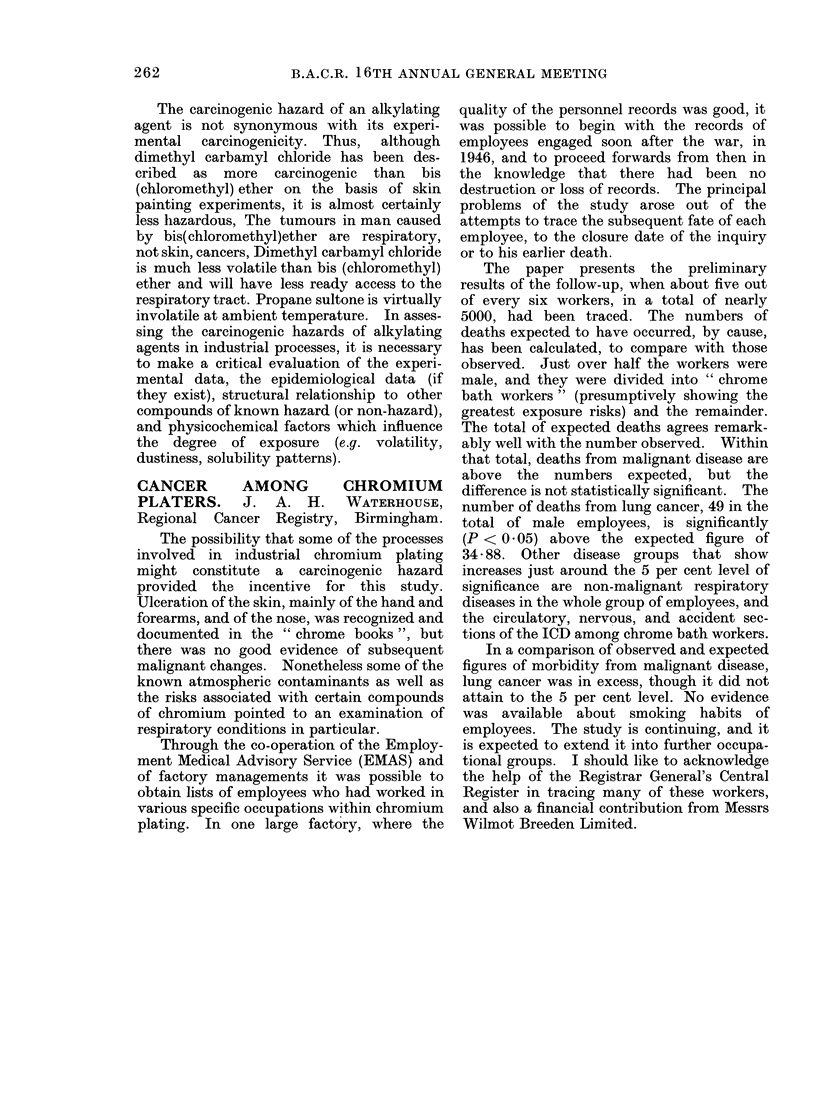# Proceedings: Alkylating agents as industrial carcinogens.

**DOI:** 10.1038/bjc.1975.210

**Published:** 1975-08

**Authors:** A. Munn


**ALKYLATING AGENTS AS INDUSTRIAL CARCINOGENS.** A. Munn, Imperial Chemical Industries Ltd.

This paper is concerned primarily with alkylating agents used in industrial processes rather than with alkylating agents used only as laboratory reagents, or with therapeutic agents requiring metabolic processing to enable biological alkylation to take place.

The experimental carcinogenicity of bis (chloromethyl) ether, chloromethyl methyl ether, dimethyl carbamyl chloride, dimethyl sulphate, propane sultone, betapropiolactone is reviewed and it is pointed out that butane sultone and gammabutyrolactone are inactive (or weakly active at worst).

Whereas the hazard to man of bis (chloromethyl) ether and chloromethyl methyl ether has been demonstrated by epidemiological data, the data for dimethyl sulphate are inadequate, and for the other compounds mentioned above there are no epidemiological data. The position in respect of dimethyl sulphate demands urgent clarification and it is hoped that the new Health and Safety Commission will conduct an epidemiological survey in respect of the only major manufacturing plant in the United Kingdom.

Carcinogenic N-nitrosamines require metabolic transformation. They have scarcely been used in industrial processes in the U.K., although they may be inadvertently generated where secondary amines (*e.g.* morpholine) are brought together with nitrite, as may occur in some cutting oils. While both N-nitroso-diphenylamine and dinitrosopentamethylene tetramine have been used industrially on a substantial scale, experimental investigation indicates that neither is carcinogenic.

The carcinogenic hazard of an alkylating agent is not synonymous with its experimental carcinogenicity. Thus, although dimethyl carbamyl chloride has been described as more carcinogenic than bis (chloromethyl) ether on the basis of skin painting experiments, it is almost certainly less hazardous, The tumours in man caused by bis(chloromethyl)ether are respiratory, not skin, cancers, Dimethyl carbamyl chloride is much less volatile than bis (chloromethyl) ether and will have less ready access to the respiratory tract. Propane sultone is virtually involatile at ambient temperature. In assessing the carcinogenic hazards of alkylating agents in industrial processes, it is necessary to make a critical evaluation of the experimental data, the epidemiological data (if they exist), structural relationship to other compounds of known hazard (or non-hazard), and physicochemical factors which influence the degree of exposure (*e.g.* volatility, dustiness, solubility patterns).